# Supplementary material for: MetaRibo-Seq measures translation in microbiomes
Source: Nat Commun. 2020 Jun 29;11:3268. doi: 10.1038/s41467-020-17081-z (PMC7324362; doi:10.1038/s41467-020-17081-z)
Supplement: Supplementary file 10 — Supplementary Data 7 [file 41467_2020_17081_MOESM10_ESM.zip › File2/Confidence_High_Taxonomy/59218_out.krona.html]

Javascript must be enabled to view this page.

members
magnitude
magnitudeUnassigned
count
unassigned
taxon
rank

59218\_out

3


SRS013687\_contig\_number\_20210
1

2
superkingdom
2759

2
kingdom
4751

2
subkingdom
451864

4890
phylum
2

147537
2
subphylum

4891
class
2

4892

SRS051031\_contig\_number\_contig-100\_2121.196980SRS1041145\_contig\_number\_contig-100\_1930.186003
order
2
